# Study Design and Rationale for the Phase 3 Clinical Development Program of Enobosarm, a Selective Androgen Receptor Modulator, for the Prevention and Treatment of Muscle Wasting in Cancer Patients (POWER Trials)

**DOI:** 10.1007/s11912-016-0522-0

**Published:** 2016-05-02

**Authors:** Jeffrey Crawford, Carla M. M. Prado, Mary Ann Johnston, Richard J. Gralla, Ryan P. Taylor, Michael L. Hancock, James T. Dalton

**Affiliations:** Duke Cancer Institute, Duke University, Duke University Medical Center, 441 Seeley G. Mudd Building, 10 Bryan Searle Drive, Box 3476, Durham, NC 27710 UK; Department of Agricultural, Food and Nutritional Science, University of Alberta, 4-002 Li Ka Shing Centre, Edmonton, AB T6G 2P5 Canada; GTx Inc., 175 Toyota Plaza, 7th Floor, Memphis, TN 38103 USA; Albert Einstein College of Medicine, Jacobi Medical Center, 1400 Pelham Parkway South, Building 1, Room 3N20, Bronx, NY 10461 USA; University of Michigan, College of Pharmacy, 428 Church Street, Ann Arbor, MI 48109 USA

**Keywords:** Muscle wasting, Sarcopenia, Selective androgen receptor modulator, Cachexia, Non-small-cell lung cancer, Enobosarm, Cachexia treatment, Cachexia therapy

## Abstract

Muscle wasting in cancer is a common and often occult condition that can occur prior to overt signs of weight loss and before a clinical diagnosis of cachexia can be made. Muscle wasting in cancer is an important and independent predictor of progressive functional impairment, decreased quality of life, and increased mortality. Although several therapeutic agents are currently in development for the treatment of muscle wasting or cachexia in cancer, the majority of these agents do not directly inhibit muscle loss. Selective androgen receptor modulators (SARMs) have the potential to increase lean body mass (LBM) and hence muscle mass, without the untoward side effects seen with traditional anabolic agents. Enobosarm, a nonsteroidal SARM, is an agent in clinical development for prevention and treatment of muscle wasting in patients with cancer (POWER 1 and 2 trials). The POWER trials are two identically designed randomized, double-blind, placebo-controlled, multicenter, and multinational phase 3 trials to assess the efficacy of enobosarm for the prevention and treatment of muscle wasting in subjects initiating first-line chemotherapy for non-small-cell lung cancer (NSCLC). To assess enobosarm’s effect on both prevention and treatment of muscle wasting, no minimum weight loss is required. These pivotal trials have pioneered the methodological and regulatory fields exploring a therapeutic agent for cancer-associated muscle wasting, a process hereby described. In each POWER trial, subjects will receive placebo (*n* = 150) or enobosarm 3 mg (*n* = 150) orally once daily for 147 days. Physical function, assessed as stair climb power (SCP), and LBM, assessed by dual-energy X-ray absorptiometry (DXA), are the co-primary efficacy endpoints in both trials assessed at day 84. Based on extensive feedback from the US Food and Drug Administration (FDA), the co-primary endpoints will be analyzed as a responder analysis. To be considered a physical function responder, a subject must have ≥10 % improvement in physical function compared to baseline. To meet the definition of response on LBM, a subject must have demonstrated no loss of LBM compared with baseline. Secondary endpoints include durability of response assessed at day 147 in those responding at day 84. A combined overall survival analysis for both studies is considered a key secondary safety endpoint. The POWER trials design was established with extensive clinical input and collaboration with regulatory agencies. The efficacy endpoints are a result of this feedback and discussion of the threshold for clinical benefit in patients at risk for muscle wasting. Full results from these studies will soon be published and will further guide the development of future anabolic trials. Clinical Trial ID: NCT01355484. https://clinicaltrials.gov/ct2/show/NCT01355484, NCT01355497. https://clinicaltrials.gov/ct2/show/NCT01355497?term=g300505&rank=1.

## Introduction

Cancer is a disease associated with severe muscle wasting caused by a variety of neural, nutritional, pro-inflammatory, and autocrine/endocrine factors that ultimately culminate in an imbalance between anabolism and catabolism. Emerging evidence suggests that muscle wasting in cancer can be an occult condition, present in individuals with normal or even high body weight [1]. Importantly, muscle wasting in cancer is common and an independent predictor of poorer physical function, higher incidence of chemotherapy-related toxicity, shorter time to tumor progression, increased length of hospital stay, and shorter survival [1–3].

Muscle wasting in cancer occurs across tumor sites and stages and is more pronounced and prevalent in patients with advanced gastrointestinal and/or lung cancer, where cachexia is often manifested. Muscle wasting in cancer patients represents a significant unmet medical need, as at the time of diagnosis, 50 % of patients with advanced cancer will have skeletal muscle loss [4]. Furthermore, the proportion of patients experiencing muscle wasting at some point during the course of their malignancy increases to greater than 80 % [5]. In addition, evidence suggests that up to 20 % of all cancer deaths are directly caused by cachexia [6]. Muscle wasting in patients with NSCLC has been associated with decreased functional status, higher incidence of chemotherapy toxicity, and shorter survival.

When muscle wasting is accompanied by weight loss, a diagnosis of cachexia may be made. Cancer cachexia has been more recently defined as a complex syndrome characterized by ongoing skeletal muscle loss with or without an accompanying loss of fat mass [7, 8••]. This multifactorial syndrome cannot be completely reversed with conventional methods of nutritional supplementation or appetite stimulation and causes progressive functional impairment [8••].

The prevalence and significance of muscle wasting in cancer, as well as the lack of an effective treatment for this condition, underscore the importance of developing an effective therapeutic approach for the prevention and treatment of cancer-associated muscle wasting. Although megesterol acetate has been approved in some countries for cancer cachexia, the general consensus is that it has little effect on lean mass [9] and, to date, no pharmacologic treatments are approved for the prevention and treatment of muscle wasting. As muscle wasting has been primarily studied under the auspices of cancer cachexia, most appetite stimulants, anti-inflammatory and anabolic or nutritional agents have failed to effectively prevent or reverse this condition. Increasing nutritional delivery of energy may preserve or increase fat mass without increasing muscle mass, which could paradoxically be detrimental to patients [4, 10, 11•]. Some of the more successful attempts at treating cachexia-associated muscle wasting in cancer have resulted from anabolic androgenic steroids, which have been shown to increase protein synthesis and lean body mass (LBM) in wasting disorders [12–15]. However, in addition to promoting anabolism, these agents also have unfavorable androgenic effects including an increased risk of hepatic toxicity, acne, increased sebum production, and virilization and hirsutism in women, and potential concerns related to benign prostatic hyperplasia (BPH) and prostate cancer in men, that may outweigh their benefits [12, 16]. Ideally, a treatment should be able to produce anabolism selectively without the accompanying androgenic side effects, which has led to increased research on nonsteroidal, selective androgen receptor modulators, or SARMs [17].

## Need for a Novel Study Design

In contrast to the development of new chemotherapies, there are no standard methods or outcomes for studying the prevention and treatment of muscle wasting and a novel study design was needed to demonstrate the clinical benefit of any therapeutic intervention for muscle wasting. In addition to assessing the efficacy of a drug to prevent and treat muscle wasting by measuring changes in physical function and LBM, it is essential to include safety endpoints related to disease burden and survival. Although the relationship between muscle mass and physical function is still obscure (i.e., the magnitude of LBM increase which is needed to promote an increase in function), physical function endpoints that are reflective of activities of daily living may be used to determine the clinical benefit associated with preserving or increasing LBM. Quality of life (QoL) also needs to be assessed in these trials, especially in patients who demonstrate a response on the physical function assessment. Available methods that measure the impact of anorexia, fatigue, or cachexia on a patient’s quality of life include the Functional Assessment of Cancer Therapy (FACT) questionnaire and its subscales [18, 19].

## Novel Drug: Enobosarm

Enobosarm (GTx-024; GTx, Inc., Memphis, TN) is an orally bioavailable nonsteroidal SARM [20]. The potential of enobosarm for clinical utility was demonstrated by its selective activity in anabolic tissues at low doses in preclinical trials [21]. In the clinical setting, the use of enobosarm led to significant improvements in LBM and physical function in a phase 2, double-blind, placebo-controlled study in healthy postmenopausal women and elderly men [20]. In a phase 2b, double blind, placebo controlled study, enobosarm treatment was well tolerated and led to significantly improved LBM, physical function, and quality of life in men older than 45 years and postmenopausal women with non-small-cell lung cancer (NSCLC), colorectal cancer, non-Hodgkin’s lymphoma, chronic lymphocytic leukemia, or breast cancer [22••]. Androgenic adverse events, including negative effects on the prostate, virilization and hirsutism, have not been reported [20].

Building on these findings, the phase 3 *P*revention and treatment *O*f muscle *W*asting in patients with canc*ER* (POWER) studies (POWER 1 and POWER 2) are designed to assess the efficacy and safety of enobosarm for the prevention and treatment of muscle wasting in patients undergoing first-line chemotherapy for NSCLC, with the ultimate goal of demonstrating the clinical benefit of enobosarm on physical function and LBM. Consultation occurred with several agencies: the US Food and Drug Administration (FDA), the Medicines and Healthcare Products Regulatory Agency (MHRA; UK), and the Medical Products Agency (MPA; Sweden), at different stages of the study development. MHRA and MPA were consulted after the protocol was finished but before the study’s standard operation procedure (SAP) was written. Consultation with them was ultimately responsible for the inclusion of the continuous variable analyses.

## Design of the POWER Trials

As mentioned above, two identical trials were designed to evaluate the efficacy and safety of enobosarm for the prevention and treatment of muscle wasting in patients with NSCLC undergoing first-line platinum-based chemotherapy either with a taxane (POWER 1) or non-taxane (POWER 2). A range of potential standard chemotherapy regimens allow the treatment of the study population to reflect standard of care at the community level, including the most common chemotherapy in this setting. Additionally, similar outcomes were expected across these chemotherapy regimens in terms of toxicity and clinical response within each trial, reducing potential heterogeneity associated with treatment within the taxane trial and within the non-taxane trial. First-line treatment with tyrosine kinase inhibitors is prohibited in these studies to maintain a homogenous patient population in terms of first-line chemotherapy and avoid any potential concerns related to the ability of the tyrosine kinase inhibitors to exacerbate muscle wasting [23]. Importantly, this exclusion criterion allows study participation by the majority of patients with NSCLC undergoing first-line treatment and allows subjects to receive tyrosine kinase inhibitors if clinically warranted after potential tumor progression during the trials (failed first-line chemotherapy).

As muscle wasting has multifactorial etiologies that differ depending on the type of malignancy with which it is associated (pancreatic cancer vs head and neck cancer vs esophageal cancer, as examples), the success of a phase 3 clinical trial depends on further limiting heterogeneity by studying one specific tumor type at a time. NSCLC was chosen as a representative cancer for these phase 3 studies primarily because lung cancer is the leading cause of cancer death in the western world, including the USA [24] and up to 85–90 % of lung cancer cases are NSCLC [25]. There were approximately 1.8 million new cases of lung cancer reported worldwide in 2012 [26]. Approximately 50 % of patients with NSCLC and greater than 60 % of men with NSCLC have already developed severe muscle wasting by the time their malignancy is diagnosed [4]. Moreover, in the preceding phase 2b trial of enobosarm in patients with NSCLC, significant losses in LBM occurred over the course of the study (4 months) in the placebo arm, while enobosarm improved physical function and LBM.

Importantly, the FDA is in agreement with including NSCLC as an appropriate cancer type to target in the phase 3 trial as these patients would likely present with a median survival of a sufficient duration to measure the effect of the therapy. As other cancer types are associated with more aggressive muscle wasting (e.g., pancreatic cancer), the shorter overall survival represents a potential challenge for a 5-month intervention.

### Patients

The full inclusion and exclusion criteria for POWER 1 and POWER 2 can be found in Table 1. In short, postmenopausal females and males >45 years of age with a diagnosis of stage III or IV NSCLC prior to the initiation of first-line chemotherapy are eligible. Subjects do not have to meet a minimum or maximum amount of weight loss at baseline to be eligible for the study. This allows the inclusion of patients with and without muscle wasting prior to study entry, allowing for the evaluation of both prevention and treatment of this condition. The trial was conducted in 60 academic and experienced community sites in Eastern Europe, South America, and North America.

**Table 1:** POWER 1 and POWER 2 inclusion and exclusion criteria

Inclusion	Exclusion
•Voluntary, signed informed consent •BMI ≤32 and weight <300 lb (<136 kg) •Diagnosis of stage III or IV NSCLC •First-line chemotherapy not yet started •Planned first-line chemotherapy regimen is platinum plus a taxane (POWER 1) or platinum plus a non-taxane agent (POWER 2) •Screening to occur ≥4 weeks (28 days) postsurgery (in surgical cases) •Life expectancy >6 months •ECOG score ≤1 •Serum creatinine ≤2.0 mg/dL •Men: aged ≥30 years •Women: aged ≥30 years with clinical confirmation of postmenopausal status •Men: agree to use a double-barrier method of contraception during and 3 months after study •Men: serum PSA ≤4.0 ng/mL or biopsy negative for prostate cancer within 6 months	•Clinically significant concurrent illness that would interfere with protocol compliance or follow-up (investigator judgment) •ALT/SGPT or AST/SGOT >1.5 times the ULN without and >5 times the ULN, evidence of liver metastases •Alkaline phosphatase >3 times the ULN and/or total bilirubin >2 mg/dL at baseline •Biologic agents or kinase inhibitors in chemotherapy regimen including, but not limited to bevacizumab, gefitinib, and erlotinib •Uncontrolled hypertension, congestive heart failure, or angina •Stage 4 chronic obstructive pulmonary disease •HBsAg positive for hepatitis B, unless diagnosis was >10 years prior to enrollment with no evidence of active liver disease •Positive screen for hepatitis C antibody, hepatitis A antibody IgM, or HIV •Use of testosterone, oxandrolone, testosterone-like androgenic agents, or antiandrogens within 30 days of study (requiring permission in cases of long-term depot within 6 months) •Current use of megestrol acetate, dronabinol, medical marijuana, or any prescription medication intended to increase appetite or treat unintentional weight loss •Baseline stair climb time ≥30 s (mean of two stair climb tests) •Active cancer, other than NSCLC or nonmelanoma carcinoma of the skin, within the past 2 years

*ALT* alanine aminotransferase, *AST* aspartate aminotransferase, *BMI* body mass index, *ECOG* Eastern Cooperative Oncology Group, *HBsAg* hepatitis B surface antigen, *IgM* immunoglobulin M, *NSCLC* non-small cell lung cancer, *POWER* Prevention and treatment Of muscle Wasting in patients with cancER, *PSA* prostate-specific antigen, *SGOT* serum glutamic oxaloacetic transaminase, *SGPT* serum glutamic pyruvic transaminase, *ULN* upper limit of normal

### Overall Study Design

**Fig. 1:**
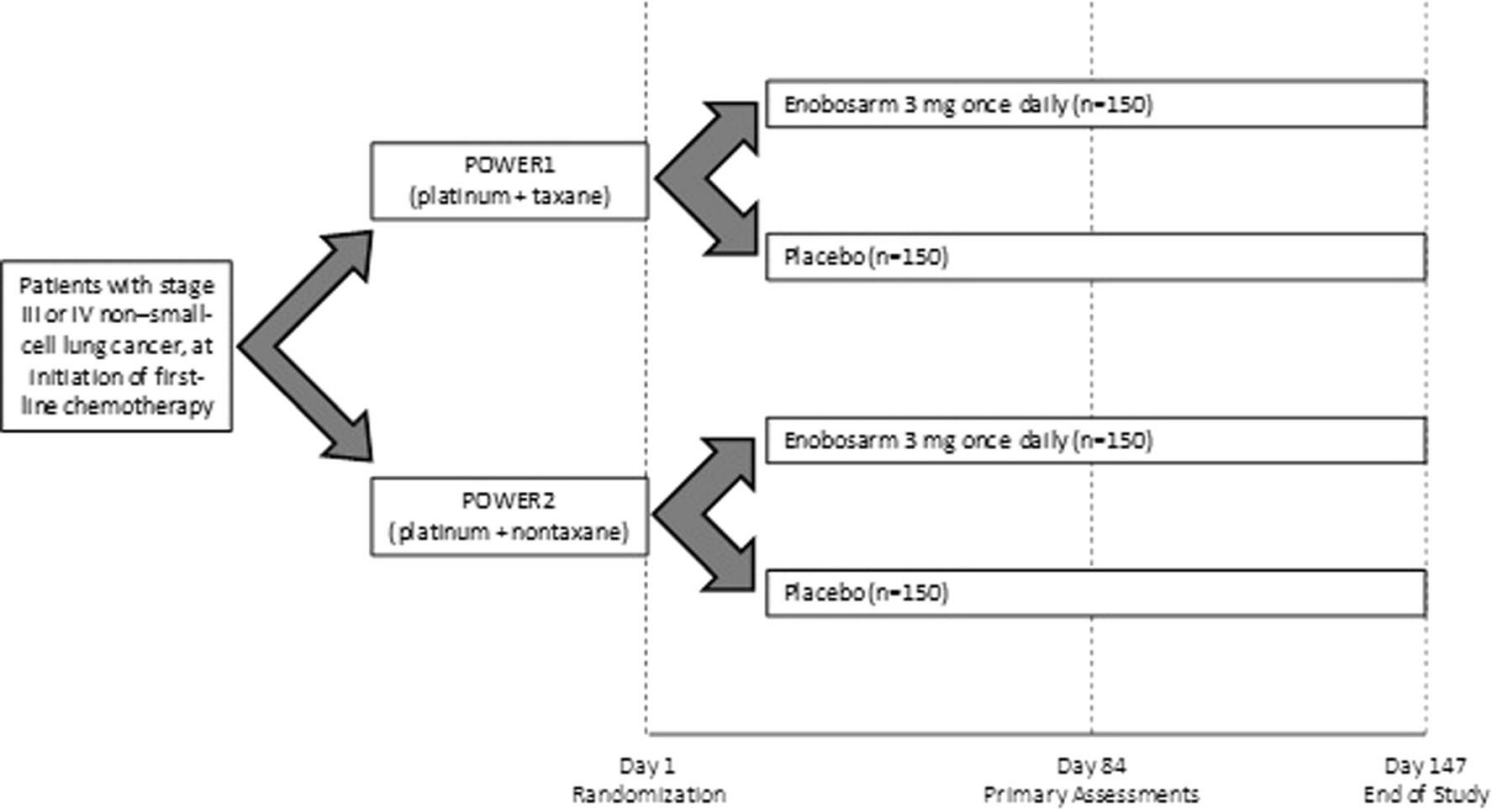
POWER 1 and POWER 2 study design

POWER 1 and POWER 2 are identically designed, phase 3, randomized, double-blind, placebo-controlled, multicenter, multinational trials (Fig. 1). A total of 300 patients are to be enrolled into each study (see statistical section for details). Randomization is as follows: 1:1 ratio to either placebo (*n* = 150) or enobosarm 3 mg (*n* = 150), with patients stratified to balance the distribution of first-line chemotherapy regimens, gender, and NSCLC stage (III vs IV). Study treatment will be administered orally once a day for up to 147 days, with follow-up of only survival continuing after day 147.

### Endpoints and Justification for Response Criteria

A comprehensive list of all efficacy and safety endpoints can be found in Table 2. The *co*-*primary endpoints* were physical function and LBM response assessed at baseline, day-42, day-84, and day-147 visits. A patient is deemed to have a physical function response if a ≥10 % increase in stair climb power at the day-84 visit is observed as compared with baseline. This threshold was established based on clinical meaningfulness from previous literature [27, 28]. A patient is deemed to have had a LBM response if their LBM at the day-84 visit is the same as or higher than their LBM at baseline.

**Table 2:** POWER 1 and POWER 2 endpoints

Key	Study day
US Food and Drug Administration: coprimary	
10 % Increase in physical function via stair climb power	84
No loss of total LBM	84
MHRA/MPA: primary and key secondary	
Change in physical function via stair climb power; continuous variable analysis	
Change in total LBM; continuous variable analysis	
Secondary	Study day
Physical function via stair climb power (day 84 responders only)	147
Total LBM (day 84 responders only)	147
Change in stair climb power from baseline^a^	84
Change in LBM from baseline^a^	84
Overall survival (data pooled from both studies)	N/A
Tertiary	Study day(s)
Healthcare resource utilization	0, 21, 42, 63, 84, 105, 126, 147
Percentage of adherence to baseline chemotherapy regimen^b^	21, 42, 63, 84, 105, 126, 147
Change in total body weight	0, 21, 42, 63, 84, 105, 126, 147
QOL via FAACT-12®	0, 42, 84, 147
QOL via FACIT Fatigue Scale®	0, 42, 84, 147
QOL via PROMIS® Physical Functioning Short Form 10a	0, 42, 84, 147
QOL via PROMIS® Emotional Distress-Depression Short Form 8b	0, 42, 84, 147
QOL via EQ-5D-5 L™	0, 42, 84, 147
5 % Increase in physical function via stair climb power	84
Chemotherapy tolerability	21, 42, 63, 84, 105, 126, 147
Safety	Study days
Routine safety assessments (adverse events, chemistries, urinalysis^c^, hematology)	0, 21, 42, 63, 84, 105, 126, 147
Overall survival	N/A
Serum hormone levels	0, 84, 147
Hair growth (women)	0, 84, 147
Serum PSA levels (men)	0, 21, 42, 63, 84, 105, 126, 147
ECOG status	84, 147
Tumor progression by RECIST 1.1 classification	84, 147

*MHRA* Medicines and Healthcare Products Regulatory Agency, *MPA* Medical Products Agency, *ECOG* Eastern Cooperative Oncology Group, *FAACT* Functional Assessment of Anorexia and Cachexia Therapies, *FACIT* Functional Assessment of Chronic Illness Therapy, *QOL* quality of life, *PROMIS* Patient-Reported Outcomes Measurement Information System, *PSA* prostate-specific antigen, *RECIST* Response Evaluation Criteria In Solid Tumors

^a^Mean change to be calculated for each treatment arm

^b^Change from baseline includes initiation of second-line chemotherapy regimen

^c^Routine urinalysis was performed only on days 0, 84, and 147

### Rationale for Physical Function Tests

Physical function tests have been utilized in the approval of medications to treat diseases associated with functional limitations such as multiple sclerosis, pulmonary arterial hypertension, and HIV-associated wasting [29–31].

The stair climb test was chosen for these trials based on its association with everyday living and is associated with strength, balance, mobility, speed, and endurance [32]. The stair climb power has been a physical function method of choice on previously conducted clinical studies in populations with or at risk for muscle loss (including NSCLC patients). It is a simple and safe measure associated with measures of lower-limb muscle strength and power and functional performance in older adults [33]. Decreases in stair climb power in elderly patients have been associated with detrimental changes in balance and falls and morbidity and mortality, whereas increases have been associated with improvements in QOL [32].

Stair climb power is calculated as power (watts) = work/time = force × velocity. In addition to strength, it takes into consideration a constellation of muscle-related attributes including balance, mobility, and endurance [32]. Due to the level of physical intensity required to climb stairs, increases in stair climb power should equate to similar or greater improvements in other less physically intense daily activities that are either short in duration or utilize smaller muscle groups (i.e., walking a short distance, rising to a standing position from a chair, or lifting or carrying household items). Furthermore, the stair climb test is a direct measure and is a well-accepted, reproducible, portable, and objective measure of physical function [32].

Regardless of the physical function test used, thresholds of clinically meaningful change have been established. A minimally clinically meaningful change in physical function is a 5 % increase from baseline and a substantial clinically meaningful change is a 10 % increase from baseline. Published literature in healthy elderly and mobility limited subjects has correlated measures of physical function with clinically meaningful changes as established in the Short Physical Performance Battery (SPPB). In a large randomized trial with adults aged 70–89 (*N* = 424) Kwon et al. utilized 400-m walk and gait speed and demonstrated that a 4–4.5 % improvement in physical function translates into “minimally meaningful” change while an improvement of 10 % represents a “substantial meaningful” change [27]. Perera et al. showed similar results with gait speed, 6-min walk distance, and self-reported mobility in older adults with mobility disabilities (*N* = 492) concluding that an improvement in performance of 6–8 % represents a “small meaningful change” and 11–17 % a “substantial meaningful change” [28]. These studies define thresholds for “minimally meaningful” and “substantial meaningful” clinical change that can be applied regardless of the physical function test used.

### Rationale for Body Composition Assessment

Although CT, MRI, and DXA are the methods of choice for the assessment of lean and adipose tissue compartments [34–39], DXA was selected as a method of choice for the POWER trials due to its reliability and availability in clinical settings (typically used for the assessment of osteoporosis and its cost-efficiency with minimal radiation exposure) [40, 41]. Additionally, DXA measures are strongly correlated with the state-of-the-art CT or MRI [42–46]. Since skeletal muscle is such an important component of the LBM compartment, changes in DXA-assessed LBM reflect changes in skeletal muscle mass [39, 47].

LBM response is defined as no loss in LBM at the day-84 visit compared to baseline. DXA scans of the whole body are performed at approximately the same time of day and by the same technician, with the patient in a similar position using the same DXA scanner and software version. DXA scans will be read centrally by Synarc Imaging (www.synarc.com).

### Additional Assessments

CT scans will also be performed at the days-84 and day-147 visits to assess tumor status, using contrast of the chest and abdomen, including the liver and adrenal glands. If subjects have brain metastases at baseline, CT or MRI will also be assessed at these time points.

Quality of life will be assessed using five different tools: the Functional Assessment of Anorexia and Cachexia Therapies (FAACT-12®), the Functional Assessment of Chronic Illness Therapy–Fatigue Scale (FACIT Fatigue Scale®), Patient-Reported Outcomes Measurement Information System (PROMIS®) Physical Functioning Short Form 10a, PROMIS® Emotional Distress-Depression Short Form 8b, and the EQ-5D-5 L™ (Table 2). These QoL instruments were intended to provide insights regarding how the patients’ health, physical functioning, and ability to care for themselves have been affected by their disease, as well as their level of emotional distress and fatigue and perceptions about the importance of various disease characteristics.

### Statistical Methods

The two trials differ only in the choices of chemotherapy. Both trials will evaluate the same endpoints of lean body mass and stair climb power. After discussion with US and European regulatory authorities, it was decided that different methods of analysis of the SCP and LBM end points would be used in the two regions.

For the US authorities, a responder analysis will be performed for LBM and SCP as coprimary endpoints.

For European authorities, SCP will be the primary endpoint and LBM secondary. Both will be analyzed by longitudinal analysis of percent change from baseline through days 84 and 147. Both analyses have different strengths and weaknesses, but will be complementary to understanding the treatment outcomes for enobosarm in the population and are further described below.

The design proposed to US regulatory authorities is a responder analysis consisting of co-primary end points, one for physical function and one for LBM. Physical function response is defined as ≥10 % improvement from baseline to day 84, and LBM response is defined as no loss of LBM from baseline to day 84. Non-response is a failure to meet the response definition or not having the day-84 assessment for any reason. Missing data is accounted for by this definition of non-response. The design assumes a proportion of response among treated subjects of 0.20 above the control response proportion for each of the physical function and LBM endpoints. Retrospective application of the response definitions to the subset of NSCLC subjects in the predecessor phase 2b trial showed the maximum control response was 25 % for the LBM endpoint (19 % for SCP); this was inflated to 30 %, that the difference in the proportions responding to both end points was approximately 0.20, and with other parameters α = 0.05 and power = 90 %, the sample size required was 124 subjects per arm. Built into the computation above was the assumption that 30 % of subjects would be considered non-responders due to missing the day 84 primary endpoint assessment. FDA requested that the sample size be increased to 150 subjects per arm for the purposes of the safety data base. The 0.20 difference in proportion of response between the two arms covers a wide range of possible control response proportions so that for control, response proportions from 0.20 to 0.75 all have power >90 % to detect a 0.20 difference at *α* = 0.05 with 150 subjects per arm. Specifically, at the aforementioned 0.30 control, response power is 93.3 %. Overall study success for US purposes is defined as rejecting the null hypothesis for both primary endpoints using a two-sided type I error probability of 0.05 for each. Considering the need for both co-primaries to be statistically significant, the power for each trial is at least 86.5 % under the assumption of no correlation between endpoints.

Each endpoint will be tested separately for significance using a Monte Carlo approximation to the exact Cochran-Mantel-Haenszel test stratified by chemotherapy regimen (platinum plus paclitaxel or platinum plus docetaxel for POWER 1 and platinum plus gemcitabine or platinum plus pemetrexed or platinum plus vinorelbine for POWER 2), gender, and cancer stage (III or IV). Importantly, a patient may be a responder for one endpoint but not the other. Because all subjects randomized and treated will have a response classification, the primary analysis is intent-to-treat.

Analyses associated with the continuous form of the data, rather than the dichotomous form of the data used in the responder analyses, will be used to analyze secondary endpoints. As noted, the percentage change in power will be the sole primary endpoint (stair climb power) for European regulatory authorities. Random coefficients models (RCM)—also termed mixed model repeated measures (MMRM) analyses—will be used in order to include all available data, including the day 42 assessment, for the physical function endpoint including replicates, study day of assessment, treatment arm, and the interaction between treatment arm and study day of assessment. A significant interaction (*p* < 0.05) would indicate significantly different slopes (rates of change per day) between the enobosarm-treated arm and the placebo arm. The same methodology will be applied to the continuous form of the LBM data; however, there are not replicates of DXA results at each time point, unlike the physical function test. For each of the physical function and LBM endpoints, an MMRM analysis that compares the mean of post-baseline measures between the two arms will be undertaken as well. Hochberg’s methodology for controlling alpha will be applied, so that, e.g., if the slope analysis are not significant for say physical function, testing will move to the post-baseline mean analysis but the alpha level required for a significant result will be *α* = 0.025, and the physical function endpoint will be considered significant. Hierarchical testing will be used to control alpha across all of the secondary endpoints so that if an endpoint is deemed non-significant, i.e., both the slope and post-baseline mean analyses are non-significant, formal statistical testing will halt, and all further secondary endpoints below that endpoint in the hierarchy will be considered non-significant.

Responder analyses will be undertaken for QoL instruments that have individual changes that are considered a response, as already described for the coprimary endpoints. For quality-of-life instruments without a defined response, appropriate parametric or nonparametric tests will be used to compare differences in distributions. Additional sensitivity and subgroup analyses are planned.

Survival analysis will be conducted as a predefined safety endpoint of the clinical program to ensure that there was no detrimental effect of enobosarm on the underlying cancer. Additionally, the survival outcome data will be pooled to formally assess superior survival in the enobosarm group compared with placebo. The total number of patients expected for the survival assessment is 600; the survival assessment is event-driven and will require at least 450 deaths. Survival will be estimated by the Kaplan–Meier method; differences in survival distributions will be compared with a stratified log-rank test, stratified by chemotherapy (taxane, non-taxane; effectively stratifying by trial), sex, and stage. It is projected that the 450 deaths will be realized at approximately 2.84 years after accrual of the first patient. Although the trials are not prospectively powered to detect a survival difference, if median survival in the combined placebo arm is assumed to be 1 year and uniform accrual of all 600 patients occurs in 1 year (both trials starting at approximately the same time), then the test would have 86.6 % power to detect a hazard ratio of ≤0.75. The actual critical hazard ratio estimate of ≤0.831 would be associated with a significant (*p* < 0.05) result and lead to a conclusion of survival superiority.

## Discussion and Conclusions

In light of the profound unmet medical need for the prevention and treatment of muscle wasting in patients with cancer, a number of agents, including SARMs, are under development for patients with cancer who are prone to muscle wasting and cachexia. The POWER trials for enobosarm are the first phase 3 clinical trials to assess a SARM for muscle wasting in patients with cancer and have addressed the need for novel study design(s) with relevant endpoints to establish meaningful clinical benefit for patients. Results from the POWER trials will soon be released and will provide the first evidence collected prospectively on the natural history of muscle wasting in patients receiving first-line chemotherapy and the magnitude and impact of a SARM used for the treatment of muscle wasting in NSCLC. Furthermore, results from the trials will be used to critically evaluate the hereby described study design.

POWER 1 and POWER 2 include subjects with no minimum or maximum amount of weight loss at baseline so that both prevention and treatment of muscle wasting can be assessed as well as the prevalence of cachexia among these patients according to recently proposed diagnostic criteria. The majority of clinical trials investigating treatments for muscle wasting or cachexia specify a minimum amount of weight loss at baseline, limiting their ability to assess the preventive potential of a specific regimen [13–15, 31, 48–52]. The importance of prevention is underscored by evidence that muscle wasting begins before the manifestation of any outward clinical signs or symptoms, including overt weight loss [53]. There is evidence to suggest that proteasome activity (an indicator of protein degradation) was increased in muscle biopsies taken from patients with gastric cancer, including patients who had not yet exhibited weight loss [54].

In addition to studying subjects who have not yet lost weight, it is also important to characterize any changes in weight that do occur throughout a study. Weight loss or gain can reflect changes in adipose tissue, skeletal muscle, or both; therefore, in order to determine whether an anabolic therapy is affecting muscle wasting, it is necessary to measure changes in body composition (i.e., LBM) at baseline and during treatment (7, 8). As CT scans have been previously acquired as part of the medical diagnosis/treatment (as discussed previously), an additional analysis of body composition by this technique is being planned [55].

An additional aspect to be evaluated in the POWER trials was the choice of functional assessments; including the stair climb test and a variety of patient-reported mental, emotional, social, and physical well-being instruments. Although defined by loss in muscle mass, much of the burden of cachexia is due to loss of function that is reflected in poor outcomes on the aforementioned functional parameters. A palliative approach addresses these by focusing on both the disease and symptoms caused by the disease, within a patient/family-focused model [56]. For this reason, the POWER trials incorporate a wide variety of tests of functioning and QoL—such as stair climb power and five separate QoL tools—to illuminate the full range of each cancer patient’s experience in light of cancer-driven cachexia [18, 19]. In the phase 2b study of enobosarm, LBM increased significantly in the 1- and 3-mg treatment groups; stair climb results also improved from baseline, by 18.0 % in the 1-mg arm and by 21.7 % in the 3-mg arm [22••]. The use of coprimary endpoints related to LBM and stair climb performance in the POWER studies may allow more definitive analysis of the relationship between these two measures.

It has been shown that initiating palliative care measures early during cancer disease trajectory can result in prolonged survival and clinically meaningful improvements in QoL and mood in patients with advanced NSCLC [57]. While the primary objectives of the POWER studies deal with body composition and physical function, tumor progression and overall survival data will also be of great interest. Although enobosarm is being studied for the prevention and treatment of muscle wasting, the potential interplay between muscle wasting and disease progression may be of interest in these trials and in future supporting studies [58].

The POWER trials include patients with NSCLC as the first population for treatment with enobosarm, but patients with other cancer types may also benefit from enobosarm for muscle wasting, which awaits confirmation in additional trials. Cancer is a highly catabolic condition and typically more severely manifested in colorectal, gastrointestinal, and pancreatic cancers even prior to the initiation of chemotherapy [59], making patients with these tumor types of particular interest for future muscle wasting and cachexia trials.

While the design of the POWER trials assesses the ability of enobosarm to prevent and treat muscle wasting in a population being treated with standard chemotherapy, future research may be warranted to include patients using newer, more targeted therapies. For example, with the approval of several tyrosine kinase inhibitors, in lung cancer, renal cell carcinoma, and other malignancies, it remains to be determined whether a SARM can benefit patients receiving these treatments. Furthermore, it has been found that these targeted cancer treatments themselves may be associated with muscle wasting, so carefully controlled studies in these settings will be needed [23].

As research in muscle wasting disorders progresses, we may see the advent of combination therapy regimens. Since there are both hypoanabolic and hypercatabolic processes at work in cachexia in a multifactorial fashion, it may be prudent to study the effects of combining an anabolic agent with an antagonist of catabolic mediators [60]. Given that the best outcome would be prevention of muscle wasting, and hence cancer cachexia, future research should focus on both prevention and treatment of this condition. It is anticipated that outcomes from the POWER 1 and POWER 2 trials will be a critical step in not only addressing a pressing unmet medical need for additional supportive care, but also significantly informing future research directions for enobosarm and other trials. Notably, several underlying clinical conditions can result in muscle wasting with and without associated cachexia, such as acquired immune deficiency syndrome (AIDS), chronic heart failure, chronic obstructive pulmonary disease (COPD), chronic renal failure, chronic infection and sepsis, and cancer [7, 61], all of which can benefit from anabolic therapy.

## POWER 1 and POWER 2 Trial Status

The POWER studies were conceived and designed in 2011, and recruitment commenced the same year. Recruitment was completed in 2013, with results being prepared for publication.
